# Looking back and moving forward in medicinal chemistry

**DOI:** 10.1038/s41467-023-39949-6

**Published:** 2023-07-19

## Abstract

Medicinal chemistry is a fast-evolving interdisciplinary research area which aims to improve human life by developing drugs to combat diseases. *Nature Communications* interviewed three scientists, Daniele Castagnolo (Associate Professor at University College London), Paramita Sarkar (postdoctoral researcher at University of Würzburg) and Dani Schulz (Director, Discovery Process Chemistry at Merck), about their careers and the past and future in medicinal chemistry research. We asked the researchers what medicinal chemistry means to them, and their opinions on the current relevance of the Rule of Five and new chemical modalities beyond the Rule of Five. We also discuss the differences between academic and industry research in medicinal chemistry and how Open Science can support collaborations for drug development.

**Figure:**
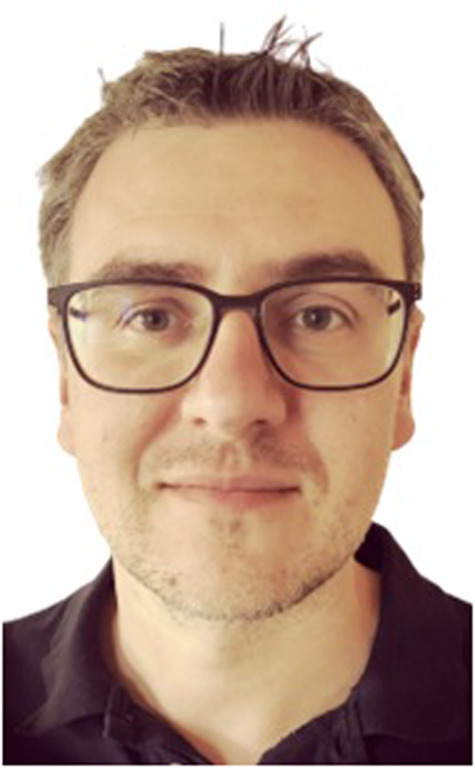
Daniele Castagnolo Daniele Castagnolo

What is your research background and how did you move into the field of medicinal chemistry research?

I became attracted to synthetic chemistry and its applications to the synthesis and design of drugs during my undergraduate studies in medicinal chemistry at the University of Siena. Such interest pushed me to continue my studies with a PhD in medicinal chemistry in the research group of Prof. Maurizio Botta, where I had a chance to work on the design and synthesis of antibacterial drugs as well as on the development of catalytic methodologies to access drugs and drug precursors through alternative and more efficient ways. During my PhD, I also started to develop an interest for organic chemistry, that led me to carry out postdoctoral studies in catalysis and organic synthesis, with Prof. Pihko in Helsinki and Prof. Clayden in Manchester. Nevertheless, I never lost sight of medicinal chemistry, so that, when I started my independent career, I decided to split my research activity into two interconnected themes: drug discovery and the development of catalytic synthetic methodologies. As a medicinal chemist, I am interested in the design and identification of new antibacterial drugs with the aim to give my contribution to the fight against antimicrobial resistance. In parallel, as an organic chemist, I like to explore novel synthetic methodologies to prepare such drugs or drug building blocks in faster, smarter and more sustainable ways than the existing ones, mainly exploiting enzymes and biocatalysis. As an example, some years ago we designed a new class of highly active pyrrole antitubercular agents and also developed a novel biocatalytic methodology to synthesise such heterocycles in a milder, greener and more sustainable way.

What is medicinal chemistry for you?

Compared to other branches of chemistry, medicinal chemistry is a highly multidisciplinary science, falling between chemistry and pharmacy and combining different disciplines. Thus, I think that it is quite hard to give an unambiguous definition of medicinal chemistry, and different scientists may have their own respectable and personal take on this. To me, doing research in medicinal chemistry means exploiting my organic chemistry knowledge to find solutions that may help to promote advancements in the treatment of a specific disease and therapeutic area. I am particularly interested in finding new synthetic routes to facilitate and accelerate the discovery of antibacterial agents to tackle antimicrobial resistance, a current global threat, by using drug hybridization strategies or recycling and repurposing drugs that have been put aside after failing pre-clinical or clinical trials.

Synthesis and modifications of bioactive, small molecular weight compounds have been a staple of medicinal chemistry in the past, while, currently, simpler-to-assemble, higher molecular weight compounds, such as targeted degradation derivatives, are receiving a lot of attention. Are we assisting at a thinking and development shift in the field, beyond Rule of 5?

One of the ultimate goals of medicinal chemistry is to develop medicines to treat a specific disease. The number of diseases affecting humans, or animals in case we work on the development of veterinary drugs, is unfortunately huge, and such diseases have diverse aetiology and biological/physiological characteristics. Treating a bacterial infection has been relatively easy in the past using the appropriate antibiotic, but a similar approach does not work against viruses, against which we normally rely on preventive vaccinations. Further different strategies are adopted in the treatment of cancer, cardiovascular diseases or even diabetes and anxiety, just to name a few. It is therefore evident that a specific disease needs a specific treatment and thus it is important for us to develop a variety of drugs for many diverse needs. Simpler-to-assemble, higher molecular weights compounds, like e.g. PROteolysis Targeting Chimeras (PROTACs), are showing promising results against cancer, and recently also against infectious diseases, but other therapeutic areas still rely on the use of small molecules. In my opinion, small molecules will continue to play a key role in the treatment of some diseases or medical conditions in the near future and thus it would be an error to state that their time is over. The more diverse therapeutic weapons we have, the better it will be. Regarding the Rule of 5, I am sure this still is and will be an important tool to design and optimise drugs. The error would be to consider the Rule of 5 an indisputable rule, and we have examples of efficient drugs going beyond it, especially natural products. However, if cleverly used, the Rule of 5 is still a very helpful tool in drug discovery and development.

In your view, which other chemical modalities are being developed and which are underdeveloped at the moment?

The recent COVID-19 pandemic showed the potential of mRNA in the preparation of vaccines against viruses. Such technology is very promising, and it could be used in the future also in cancer therapy. RNA-based drugs, like small interfering RNA (siRNA) or antisense oligonucleotides are other examples of new chemical modalities, as well as oligo- and polypeptides which are finding increasing interest and application against bacterial infections, or antibody drug conjugates and the above mentioned PROTACs in cancer therapy. Other areas which I find particularly interesting are the development of membrane disrupting agents like macrocyclic peptides, fatty acids, or lipid derivatives, especially for its implications in antimicrobial resistance, and photopharmacology, a growing area that employs photoswitchable ligands to modulate the activity of drugs.

Are there more quick and efficient screening technologies emerging that could help to speed up the drug discovery process, or to make it more affordable?

Combinatorial synthesis and diversity-oriented synthesis have been, and still are, key technologies to speed up the drug discovery processes, allowing the generation of structurally diverse hit compounds for biological assays in a relatively short time. Similarly, virtual screening, either structure-, ligand- or fragment-based, has revolutionised our way of discovering new active molecules. Recently, activity-directed synthesis has emerged as an interesting technology through which crude reaction mixtures are directly screened for biological activity. Such approach offers the advantage to enable the parallel discovery of both biologically active hit compounds and associated synthetic routes, in a manner similar to the natural evolution of biosynthetic pathways yielding natural products.

In my group, we make use of virtual screening and combinatorial techniques to identify new hit molecules. We also use the hybridization and repurposing of old and abandoned drugs, which have failed preclinical or clinical trials. The idea is to re-use such molecules by improving their pharmaceutical profile via appropriate chemical modifications. We want to develop a sustainable approach to drug discovery, minimising the drop out of hit drug candidates which still may have a biological potential, through a drug-recycling approach taking advantage of the chemical and biological data already available on these drugs, in turn compressing the time for their translation to preclinical and clinical trials. Drug hybridization is not a new drug discovery strategy and may look less efficient compared to other technologies that guarantee a more rapid generation of compound libraries. However, I like to quote Nobel laureate Sir James Black who stated that “*the most fruitful basis of the discovery of a new drug is to start with an old drug*”.

What do you think are the main differences in approach and mind-set between academia and industry?

We could define the research in academia as knowledge-centric, since one of the key aspects of academic research is the sharing of knowledge and data with the scientific community. One of the most important goals in academia is represented by the dissemination of research data through scientific publications or at conferences. Not infrequently, the research carried out in academia may have no immediate scientific or social impact, and its potential may become evident only years later. On the other hand, industry can probably be seen as more goal-centric, since its primary goal generally is, in the case of pharmaceutical industry, the development, production and commercialization of medicines and therapeutic treatments for patients. Researchers in industry focus more in developing new products (i.e. medicines) or technologies that may have an immediate impact on their clients (i.e. patients). Industries also publish and disseminate knowledge, often in collaboration with academia, but, before disclosing their discoveries to the public, they must take into account aspects such as intellectual property protection and patents. In industry, every major discovery is generally patent protected, and profitable, and any data dissemination usually follows a patent filing.

Do you think Open Science initiatives can help speed up the drug discovery process, and enable closer collaborations between industry and academia?

It is important to distinguish what can be the implications of Open Science for academia and what for industry. As an academic, I fully support Open Science initiatives, such as open access publications or sharing and dissemination of data with the public. As mentioned above, the role of academia should be mainly to share knowledge and disseminate data and discoveries. Medicinal chemists can carry out research in many areas, such as identification of new drugs, validation of new drug targets, optimization of the biological properties of drugs, development of drug-screening technologies etc. Sharing knowledge and data through Open Science can help researchers working on closely related areas to overcome problems, get new ideas and, in turn, speed up the drug discovery process. Open Science in industry is a thorny problem, since industries normally protect and patent their discoveries because they must deal with competitors and enormous cost investments, especially in clinical trials. However, there are some pharma start-ups that are already attempting to implement an open-science business model of drug development. Such models seem to work well, specifically in pre-clinical development, in the area of orphan and rare diseases, through regulatory exclusivity incentives offered by drug regulators. This could lead to closer collaborations between academia and industry in the near future, speeding up the discovery of new drugs, especially for neglected or orphan diseases like rare cancers or metabolic diseases, or tropical infections like trypanosomiasis or leprosy.

The pandemic emergency has led to more rapid than usual responses to quickly develop vaccines and medicines. What has been the biggest mind-set shift on the drug discovery process so far, in your opinion?

The recent COVID-19 pandemic has shown how the sharing of data and collaboration among researchers can lead to the development of medicines and treatments in a rapid and efficient way. The efforts made during the pandemic have been enormous in terms of human and economic resources, scheduling and planning between industries, regulatory agencies and researchers, and pace of work. Probably, such an approach is far from being sustainable in the long term and it cannot be adopted for the development of medicines. Nevertheless, the COVID-19 pandemic has given us the confirmation that collaborative, coordinated and, following off from the previous question, open science research, are and will be vital in future for successful drug discovery and development.

**Figure:**
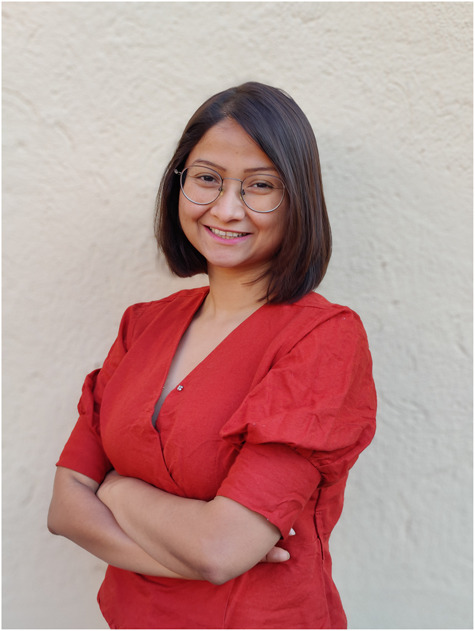
Paramita Sarkar Paramita Sarkar

What is your research background and how did you move into the field of medicinal chemistry research?

I come from the east Indian metropolitan of Kolkata where there is a passion for studying basic sciences. Partly due to the academic culture and my interest in the subject, I enrolled for Chemistry in the St. Xavier’s College (University of Calcutta) for my undergraduate degree. I was undecided about what to specialize in afterwards, but I knew I wanted to do sciences. Luckily, I got selected into a multidisciplinary integrated PhD program at the prestigious Jawaharlal Nehru Centre for Advanced Scientific Research (JNCASR, Bangalore, India). Bangalore boasts of some great scientific institutes such as the Indian Institute of Science and National Centre for Biological Sciences. This exposure to outstanding scientists attracted me to JNCASR. The programme allowed me to rotate in labs that tackled different problems at the interface of chemistry, physics, and biology. During my rotation at the lab led by Prof. Jayanta Haldar, I was exposed to the problem of antimicrobial resistance or how bacteria were evolving mechanisms to render clinically used drugs obsolete. I wondered how I could use my background in chemistry to find innovative solutions to treat multi-drug resistant bacterial infections. I started working on the modification of vancomycin. Vancomycin is an antibiotic of last resort, meaning it is used only when all other drugs fail to treat Gram-positive bacterial infections. This was my pathway to the world of medicinal chemistry and microbiology. I was fascinated by the ways in which I could introduce chemical modifications on vancomycin to make it work against bacteria that had either grown resistant or were inherently resistant to it. After completing my master’s thesis, I delved into the medicinal chemistry of glycopeptide antibiotics for my PhD. For the next four years, I would modify vancomycin to make it effective against both drug-resistant Gram-positive and Gram-negative bacteria. I then studied how the lead compounds affected the biosynthetic processes in bacteria through various phenotypic analyses, biophysical, and biochemical methods. Currently, I continue to use my skills as a medicinal chemist to develop antisense antibiotics at the Institute for Molecular Infection Biology in Würzburg, Germany.

What is medicinal chemistry for you?

Quoting Prof. Carolyn Bertozzi: “We chemists are dreamers. We think up new molecules and bring them to life”. Medicinal chemistry for me is a power to positively affect human health. I find immense pleasure in tweaking molecules with simple chemistry that leads to an improvement in their therapeutic properties. My ultimate aim is to contribute at least one drug to the clinic that will save many lives.

Synthesis and modifications of bioactive, small molecular weight compounds have been a staple of medicinal chemistry in the past, while, currently, simpler-to-assemble, higher molecular weights compounds, such as targeted degradation derivatives, are receiving a lot of attention. Are we assisting at a thinking and development shift in the field, beyond Rule of 5?

Indeed, while Lipinski’s Rule of 5 served as a starting point in medicinal chemistry, modern medicine has moved beyond small molecules. While small molecular drugs continue to be an important class of drugs, the field has advanced to newer modalities. In the last decade, quite a few drugs that are beyond the rule of 5 (bRo5) have been approved for clinical use for example, voclosporin, glecaprevir and fostamatinib. Around 50 percent of the drugs approved in 2022 are alternative therapeutics that fall in the bRo5 space. The chemical space for drugs and therapeutic strategies has expanded significantly to macromolecules such as oligonucleotide therapeutics (antisense therapy, siRNA, oligonucleotide conjugates, aptamers) and antibodies. This can largely be attributed to great technical advancements made in the field of automated chemical synthesis and biotechnology. In my opinion, for the development of potential new drugs, medicinal chemists require an understanding of the drug target and biological processes. The lines between chemistry and biology have thus become less prominent and close collaboration between the subjects is leading to a new era in drug development. For example, targeted protein degradation strategies (PROTACs), phage therapy, and CRISPR-based gene editing therapeutics, microbial therapeutics, and in vivo expressed biologics are also being developed as therapeutics. With these technologies, I believe that, in the next decade, we will see a new generation of medicines.

In your view, which other chemical modalities are being developed and which are underdeveloped at the moment?

I feel current medicine has been emerging beyond just chemical modalities. We already see a trend of synthetic biologics such as oncolytic viruses, CAR-T-cell therapy, and synthetic proteins/enzymes among others, are heralding a paradigm shift away from conventional chemical modalities. Having said that, specifically, the emerging chemical modalities being developed include macrocyclic molecules, targeted protein degraders, cyclopeptides, nanobodies, antibody-drug, and drug-drug conjugates. While significant research has been done on organic and inorganic nanoparticles and polymer-based therapeutics, they are still underrepresented in the clinics.

Are there more quick and efficient screening technologies emerging that could help to speed up the drug discovery process, or to make it more affordable?

There are several technologies that are being developed and used to speed up as well as reduce the cost of all stages of drug discovery. With the growth of artificial intelligence and machine learning, computer-aided drug discovery has revolutionized in-silico drug screening, thereby speeding up lead discovery. OMICs technologies including genomics, proteomics, and metabolomics, help in the understanding of the disease pathways and identification of drug targets. These assist medicinal chemists in designing better drugs with minimal side effects. In addition, genome-wide screening technologies using RNAi, and CRISPR-based gene editing have enabled interrogation of gene function. These help to identify new disease-causing target proteins and genes. Drug discoverers can therefore specifically screen for compounds against these new targets to speed up drug discovery. Automation and flow-based synthesis platforms that can help in rapid discovery and optimisation of small molecules have also been developed in the last decade. On the other hand, in vitro screening assays to associate signalling profiles of potential drug candidates to desired/undesired clinical outcomes are also being developed. In that regard, microfluidic lab-on-a-chip technologies can aid all stages of drug discovery, from high-throughput synthesis to drug evaluation.

What do you think are the main differences in approach and mind-set between academia and industry?

Industry and academia differ in their motivations for research and in their parameters of success. Industrial research is application-oriented with a focus on commercial potential and profitability. Academia on the other hand is more fundamental and is driven by curiosity and societal needs. This is best exemplified by the scarcity of big pharma involved in anti-infective research: due to the poor return on investment, research in this field is not as attractive and is done mostly by academic labs or small and medium-sized companies. The Global Antibiotic Research & Development Partnership, Combating Antibiotic-Resistant Bacteria Biopharmaceutical Accelerator and Drugs for Neglected Diseases initiative, among others, are examples of some initiatives that have facilitated a global partnership between private, academic, and non-profit entities to combat the challenges in anti-infective drug development.

Do you think Open Science initiatives can help speed up the drug discovery process, and enable closer collaborations between industry and academia?

I believe that Open Science initiatives are excellent to aid drug discovery. The reproducibility and translatability of preclinical findings to the clinics remain a challenge despite efforts by both academia and industry. Open Science could mitigate these issues by facilitating collaborative research and increasing transparency. The response to the COVID-19 pandemic best illustrates the benefits of open science initiatives. Publication houses such as Springer Nature and Elsevier made all COVID-related research freely accessible. Additionally, initiatives such as OpenSAFELY provided free and easy access to healthcare data thereby facilitating research on other treatment modalities. However, I believe this is the only successful example where open science has expedited the drug discovery process as much. I feel that we are still a few years away before it uproots the current model that science has adopted. For example, patent protection is fundamental for the commercial success of industrial giants. Open source is essentially anti-patent. Thus, a different commercial model needs to be adopted to include the open-source initiative.

The pandemic emergency has led to more rapid than usual responses to quickly develop vaccines and medicines. What has been the biggest mind-set shift on the drug discovery process so far, in your opinion?

The speed with which solutions were developed during the pandemic showcased the strength of open science. For me, the biggest impact that the pandemic has had on science is cross-disciplinary research towards a common goal: almost everyone doing science wanted to contribute to solving pandemic-related problems. The pandemic brought together physicists, biologists, chemists, computational scientists, statisticians, and medical doctors collaborating at a pace that we had never seen before. This led to the realisation of the need for close collaborations across fields to facilitate successful drug discovery. I believe, medicinal chemistry is in the thick of it and ties all the fields together. For example, the in-silico drug screening helps to identify interesting leads which allows medicinal chemists to synthesize homologues of the leads and quickly screen them for activity together with biologists.

**Figure:**
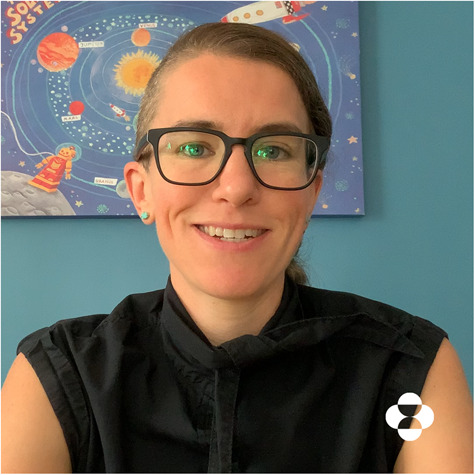
Dani Schultz Dani Schulz

What is your research background and how did you move into the field of discovery process chemistry research?

My background is in synthetic organic chemistry and my journey to where I am today at Merck & Co., Inc., USA (also known as MSD) started when I was an undergraduate at the University of Wisconsin-La Crosse. There I studied chemistry and molecular biology and was fortunate enough to be part of an undergraduate research project where I synthesized novel serotonin agonists for structure-activity relationship studies in collaboration with pharmacologists. This fascinated me for a couple reasons: first, I was observing first-hand how subtle chemical modifications can dramatically impact pharmacokinetics/pharmacodynamics (PK/PD) and second, I was creating chemical matter that had never been reported – it was quite exhilarating! My undergraduate research solidified my decision to pursue a PhD in organic chemistry at the University of Michigan with Professor John Wolfe and subsequently a postdoc at the University of Wisconsin-Madison with Professor Tehshik Yoon. As a graduate student and then postdoc, I focused on creating new synthetic methodologies with emerging technologies, such as visible-light photocatalysis, developing reactions that would rapidly assemble molecular complexity from simple precursors. Given my experience in drug discovery as an undergraduate, coupled with my training in devising efficient routes to molecules, industry seemed like the perfect fit and I joined the company in 2014 as a Senior Scientist. Since joining, I have been a member of Process Chemistry and Enabling Technologies groups, where I leveraged catalysis and high-throughput experimentation (HTE) to advance clinical candidates developed by our medicinal chemists. In 2021, I brought this experience to Discovery Process Chemistry (DPC), a group that resides at the discovery and process chemistry interface. Within this role, I lead a group of creative process chemists who apply a range of technologies to accelerate the design-make-test (DMT) cycle and advance our small molecule and peptide portfolio.

What is medicinal chemistry for you?

In my current role, I work alongside medicinal chemists and have observed first-hand their dedication to the pursuit of finding new medicines to impact human health. From that perspective, medicinal chemistry involves creativity, collaboration and a deep understanding of both biology and the interplay of the various pharmaceutical properties (potency, stability, solubility, etc.) that they are trying to optimize. For medicinal chemists, the pursuit of new medicines proceeds through the DMT cycle, designing new compounds to probe a specific biological hypothesis, synthesizing them in the lab and then testing their biological activity. In my opinion, the ‘make’ in the DMT cycle is typically rate-limiting (there are more good ideas than time to try them) and that is where DPC works alongside medicinal chemists to access chemical space where there is no established route – the ‘uncharted space.’ Partnering with medicinal chemists is incredibly exciting as we are all rowing in the same direction to find the best molecules as quickly and efficiently as possible.

Synthesis and modifications of bioactive, small molecular weight compounds have been a staple of medicinal chemistry in the past, while, currently, simpler-to-assemble, higher molecular weights compounds, such as targeted degradation derivatives, are receiving a lot of attention. Are we assisting at a thinking and development shift in the field, beyond Rule of 5?

As you have pointed out, the small molecule (SM) drug-discovery toolbox is very well established and has resulted in the small molecule modality dominating FDA approved drugs ( > 50% of the global pharmaceutical market is SMs). Beyond rule of 5 (bRo5) compounds are becoming quite prevalent within the pharmaceutical industry as they are opening the aperture of druggable space; however, unlike SMs, the toolbox to rapidly explore chemical modifications and develop bRo5 compounds comes with challenges. For instance, targeted degraders are typically composed of two small molecules (one being an E3 ligase ligand and the other being the protein of interest ligand) that are connected through a linker. While the current chemistry is fairly straightforward to stitch these three components together (i.e. amide and ether bond formations), expanding the linker toolbox and thinking holistically on new convergent methods for targeted degrader synthesis would not only accelerate drug discovery but also influence the pharmaceutical properties. Lastly, the intended route of administration for the majority of bRo5 compounds is oral delivery, yet challenges remain in optimizing the chemical matter to be stable towards gastro-intestinal proteases and with the ideal solubility/bio-physical properties for absorption and target delivery. For instance, unlike small molecules, bRo5 compounds (with molecular weights >500 Da) can readily form oligomers or higher ordered structures which can dramatically impact absorption and the overall ability to access the target. As a result, achieving optimal formulations for bRo5 compounds will be critical in the advancement of these modalities.

In your view, which other chemical modalities are being developed and which are underdeveloped at the moment?

As I mentioned above, non-naturally occurring peptides (within the bRo5 domain) are a growing therapeutic modality with most applications targeting endogenous proteins; however, advances in hit-to-lead platforms (such as mRNA display) have revealed that peptides also have the unique disposition to disrupt protein-protein interactions (PPIs) – once considered undruggable. As a result, the discovery of peptide therapeutics is rapidly evolving and there is a growing need for new chemical tools and non-canonical amino acid (ncAA) building blocks that allow the interrogation of key properties such as potency, proteolytic stability, solubility and bioavailability. In my opinion, our understanding of how best to leverage ncAAs to access a seemingly endless source of peptide chemical diversity is still in its infancy. Consequently, investments in modelling, informatics, and synthesis are needed to truly impact the future of peptide therapeutics. For example, one area that I feel is underdeveloped is methods for the late-stage functionalization (LSF) of complex peptides that are compatible both on-resin and in solution. The advantages of pursuing LSF during peptide drug discovery is quite substantial, as a single precursor peptide could be used to generate a library of boutique peptides targeting PPIs from commercial building blocks.

Are there more quick and efficient screening technologies emerging that could help to speed up the drug discovery process, or to make it more affordable?

Building off the previous question, the potential to develop therapeutic peptides to target PPIs is incredibly exciting but also incredibly daunting—as how does a drug discovery team begin to survey this vast space? Well, over the past 20 years, advances in hit finding platforms, such as mRNA display and phage display, have relieved the bottleneck of identifying biologically active peptides, hijacking well-established cellular processes to generate > 10^10 unique de novo peptides in a single round. What has been particularly exciting is that mRNA display allows the incorporation of ncAAs, greatly expanding the chemical space of peptide and protein modalities by not limiting the toolbox to the 20 canonical amino acids. However, the utilization of ncAAs in peptide drug discovery should be carefully assessed, considering both the advantages they offer in terms of the overall pharmaceutical properties of the peptide and the potential increased cost of goods that may arise when scaling the peptide during its transition into development.

What do you think are the main differences in approach and mind-set between academia and industry?

In a 2020 *Nature Chemistry* perspective that I co-authored with my colleague L.-C. Campeau titled ‘Harder, Better, Faster’ we dug into this topic and where we landed is that academia and industry have different objectives; however, we have a common goal of developing the best science *and* scientists. For academia, research groups are focused on gaining a deeper fundamental understanding of their area of study, with some interest in downstream applications. In contrast, industry is only focused on the downstream application of a drug and can be hesitant to break away from ‘tried and true’ practices to not incur delays. With that said, I believe that academia and industry can do better in breaking down the silos (or, to some, ivory towers) to share their problems to ultimately fuel innovation and hopefully adoption. We are starting to see this mind-set shift through more academic-industrial partnerships that result in exciting new chemistry geared towards ‘pharma-relevant’ problems. Specifically, these partnerships have provided new methodologies for DNA-encoded library synthesis, next generation cross-couplings that utilize inexpensive catalysts, and stereocontrolled access to diverse oligonucleosides and peptides.

Do you think Open Science initiatives can help speed up the drug discovery process, and enable closer collaborations between industry and academia?

Scientists from both academia and industry have started to engage more frequently on problem selection which is resulting in a myriad of collaborations. However, I think where Open Science initiatives shine is in the equitable access of science research to all communities, not just those that can afford it. Having a better understanding of the literature, whether you are a student, professor, or industrial scientist, can inspire and dramatically alter the trajectory of the problems that one solves. I am unsure if Open Science will enable more collaborations but I hope that it inspires students from around the world to pursue a science career thanks to lowering barriers to access scientific research, which will hopefully lead to a more diverse work force that fuels the innovation needed to accelerate drug discovery.

The pandemic emergency has led to more rapid than usual responses to quickly develop vaccines and medicines. What has been the biggest mind-set shift on the drug discovery process so far, in your opinion?

Now that we have seen how rapidly new medicines can be developed, it has made the industry think differently about how we approach drug discovery. In particular, companies’ drug discovery teams can easily become bogged down in the fear of failure, demonstrating risk aversion in the pursuit of the perfect molecule that meets all the criteria. What do I mean by this? For any discovery program, there can be several modalities under consideration or multiple indications that could be pursued in the clinic—which can diffuse efforts and slow teams down. As a result of the pandemic, you had discovery chemistry teams focusing their efforts on what mattered most, which was getting an oral anti-viral or vaccine to the market as quickly as possible. Moreover, the pandemic showed how a firm biomarker strategy and clinical trial design can truly accelerate development. Now, with the pandemic winding down, I hope drug discovery teams continue to prioritize what the patient needs focusing on identifying the best target, modality, and biomarker to move the program forward.

*This interview was conducted by Dr. Francesco Zamberlan*.

